# Dabigatran as an alternative for atrial thrombosis resistant to rivaroxaban: A case report

**DOI:** 10.1097/MD.0000000000013623

**Published:** 2018-12-21

**Authors:** Huan Sun, Qini Zhao, Yanjing Wang, Robert Lakin, Xueyan Liu, Ming Yu, Hongliang Yang, Dongmei Gao, Weiwei Chen, Guangyuan Gao, Mengjie Yan, Yuquan He, Ping Yang

**Affiliations:** a Cardiology Department, China-Japan Union Hospital of Jilin University; b Jilin Provincial Cardiovascular Research Institute; c Jilin Provincial Key Laboratory for Genetic Diagnosis of Cardiovascular Disease; d Radiology Department; e Department of Exercise Sciences, University of Toronto, Toronto, Canada; f Ultrasound Department, China-Japan Union Hospital of Jilin University, Changchun, China.

**Keywords:** anticoagulation, dabigatran, novel oral anticoagulants, rivaroxaban, thrombus

## Abstract

**Rationale:**

Anti-thrombosis therapy for atrial fibrillation (AF) management and stroke prevention is an important aspect of disease management. Novel oral anticoagulants (NOACs) are recommended by guidelines for AF management. However, if one can switch one NOAC to another when the former showed a poor effect has not been fully determined.

**Patient concerns:**

A 52-year-old man was admitted to our center for heart failure and AF with a thrombus in the left atrium.

**Diagnoses:**

Cardiomyopathy was diagnosed by cardiac magnetic resonance (CMR) and echocardiography.

**Interventions:**

He was prescribed rivaroxaban (20 mg daily) as treatment, and dabigatran (150 mg twice daily) was used when the thrombus was found to be non-response to rivaroxaban.

**Outcomes:**

The rivaroxaban did not diminish the atrial thrombus, and dabigatran was given instead which finally eliminated the thrombus.

**Lessons:**

Individualized responsiveness to NOACs should be considered and paid more attention to during clinical practice.

## 1 Introduction

Atrial fibrillation (AF) is the most common clinical arrhythmia worldwide.^[1,2]^ Anti-thrombosis therapy for AF management and stroke prevention is an important aspect of disease management,^[3]^ with non-vitamin K antagonist oral anticoagulants (NOACs) having been reported to be both safe and effective for preventing thrombus formation and stroke in AF patients.^[4]^

However, whether different NOACs are equally effective in AF treatment among patients has yet to be determined. In addition, equal recommendation of both factor IIa (i.e., Dabigatran) and factor Xa (i.e., Rivaroxaban) inhibitors among therapeutic guidelines^[3]^ further complicates optimal and individualized NOAC treatment approaches. In the present case report, we present an AF patient whose left atrial thrombus was resistant to one anticoagulant therapy but sensitive to another, which may have implications for how NOACs are prescribed in the future.

## 2 Informed consent and ethical statement

The patient signed an informed consent. The whole report meets the requirements from the institution ethical committee.

## 3 Case presentation

**Figure 1:**
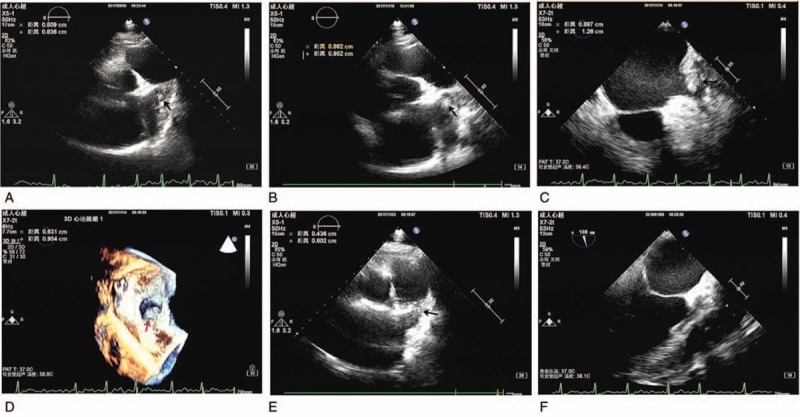
Evidence of a thrombus in the left atrium and its response to non-vitamin K antagonist oral anticoagulant (NOAC) therapy using echocardiography. (A) Transthoracic echocardiography (TTE) showed a thrombus (black arrow) in the left atrium upon initial presentation. (B) TTE imaging after 1-month rivaroxaban treatment revealed the thrombus had increased in size (black arrow). TEE imaging (C) and 3D imaging (D) further confirmed the presence of a left atrial thrombus. (E) TTE imaging showed dabigatran treatment reduced the size of the thrombus, with a later TEE examination revealing complete elimination of the thrombus (F).

A 52-year-old man was admitted to our center in late September 2017 presenting with shortness of breath as well as facial and lower limb edema. The patient was diabetic (10-year diagnosis), and was found to have had a stroke 3 months prior to his admission, resulting in slight dullness of speech. His electrocardiogram showed persistent AF (CHA2DS2-VASc score: 4). Renal function serum album levels were within normal. However, his echocardiography showed an enlarged left atrium (55 mm × 70 mm), reduced ejection fraction (44.9%), and a suspected mass (8.0 mm × 8.4 mm) in the left atrium (Fig. 1A).

**Figure 2:**
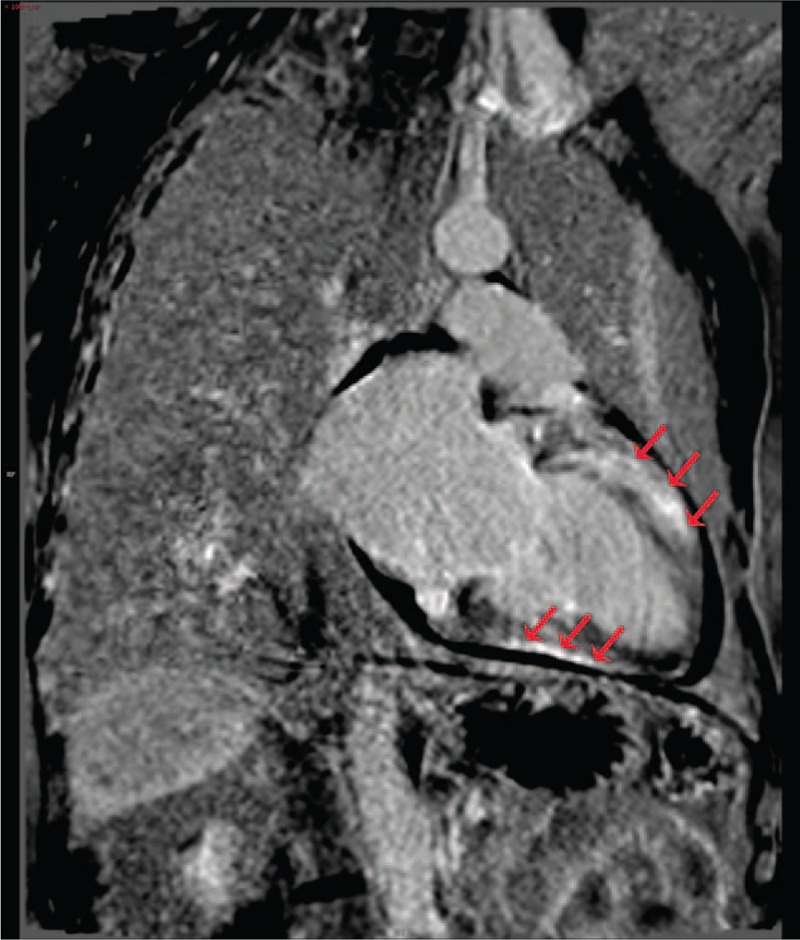
Representative cardiac magnetic resonance (CMR) image showing diffuse late gadolinium enhancement (LGE) in the left ventricle (red arrows), which implied diffuse myocardial damage in the patient.

Upon further evaluation, a normal coronary artery angiography (CAG) made the diagnosis of coronary heart disease unlikely. Subsequently, a cardiac magnetic resonance (CMR) evaluation confirmed an enlarged left atrium, with evidence of diffuse late gadolinium enhancement (LGE) in the left ventricle (Fig. 2) and a left atrial thrombus.

Based on the clinical presentation, we prescribed diuretics (furosemide 20 mg bid), ACEI (Ramipril 2.5 mg Qd), a β-blocker (metorol 49.5 mg Qd) as well as a NOAC, rivaroxaban (20 mg daily). In response to treatment, the patient's shortness of breath and edema quickly improved. After approximately 1 month (early November 2017), we performed a follow-up transthoracic echocardiography (TTE) examination that showed that the mass within the left atrium had increased in size (8.09 mm × 8.36 mm), which was further confirmed by transesophageal echocardiography (TEE). In response, we started treatment of the thrombus with a different NOAC, dabigatran (150 mg bid). During a follow-up examination in December 2017, both TTE and TEE examinations confirmed that the thrombus had decreased in size (4.36 mm × 6.02 mm) and had completely vanished in January 2018 (as shown in Fig. 1), after which AF ablation was performed to safely restore sinus rhythm. In response to its successful treatment of the thrombolytic mass, dabigatran (110 mg bid) was prescribed to this patient as a long-term strategy for stroke prevention. Following up with the patient over the next 4 months, he was free of ischemia or bleeding events.

## 4 Discussion

As thrombosis is a major complication of AF, anticoagulation therapy is essential to reduce the risk of stroke in AF patients.^[5,6]^ Previously, the RELY study^[7]^ and ROCKET-AF study^[8]^ demonstrated the efficacy of the NOACs dabigatran and rivaroxaban, respectively, for thrombosis treatment related to AF. Moreover, Lip et al^[9]^ recently reported that rivaroxaban can resolve atrial thrombi in up to 62.5% of the AF patients. Moreover, NOACs have proven to be as efficacious as warfarin in stroke prevention in AF patients, requiring less frequent monitoring of the coagulation index or adjustment of dose and making their use more convenient for physicians.^[8]^ In spite of the established benefits of NOAC treatment, it is unknown whether differences in the efficacy of these medications exist among AF patients, which would have clinical implications for the prescription and treatment success.

In the current case study, our patient initially had an unclear pathology given the lack of results of myocardial biopsy, though a diagnosis of an underlying cardiomyopathy could be made based on the CMR results. It was determined that the patient's history of diabetes was the likely cause of his myocardial injury and morphology. At this point, anticoagulation therapy and treatment for heart failure are necessary, regardless of the exact underlying pathogenesis.

It has been previously reported that rivaroxaban (10 mg daily) resolved left ventricular thrombus,^[10]^ demonstrating the high efficiency of this oral factor Xa inhibitor for thrombus treatment. By contrast, the present case demonstrated an opposite effect, with the left atrial thrombus showing evidence of enlargement after 1-month of rivaroxaban treatment. Specifically, we used rivaroxaban, at a daily dose of 20 mg, as the first choice for anticoagulation, but the evidence of thrombus enlargement led us to change our therapeutic strategy from the Xa inhibitor to dabigatran, a factor IIa inhibitor. Unexpectedly, dabigatran showed a greater efficacy in our patient, resolving the thrombus in about 1 month and proving to be superior to the Xa inhibitor in this given case. To the best of our knowledge, this is the first report to demonstrate successful thrombus breakdown with dabigatran in a patient presenting with thrombus resistance to full-dose rivaroxaban. These findings align with a recent report that showed the factor IIa inhibitor was effective at treating an atrial thrombus resistant to reduced-dose (15 mg) rivaroxaban in aged-patients.^[11]^ The reasons for the disparate responses to the NOACs used in the current study are unclear and may not be easily determined. Specifically, in spite of an overall linear dose–effect relationship for NOACs and their anticoagulant effects, this relationship may be unique for each patient, which will require an individualized approach to ensure that “super-responders” and “non-responders” are permanently identified.^[12]^ The underlying mechanism is unclear, but variability in the sensitivity of the target molecular (factor Xa and factor IIa) to specific NOACs and individualized pharmacokinetics might explain these findings, which warrants further exploration.

It should be noted that our findings do not prove that dabigatran is a superior NOAC to rivaroxaban or a Xa inhibitor in every patient presenting with a cardiac thrombus. Moreover, in support of patients showing different sensitivities to NOACs, a previous case presented a patient in which a different Xa inhibitor, apixaban, eliminated a thrombus resistant to both warfarin and dabigatran.^[13]^ Therefore, our case provides support for heterogeneity in patient responses to NOACs, which requires more attention from clinicians.

We did not perform coagulation tests for either rivaroxaban or dabigatran in our patient, which would have provided insight into the individual anticoagulation strength prior to treatment; however, this is the normal approach for NOACs prescription in AF patients. As we mentioned previously, our clinic did not perform the biopsy on this patient, which prevented us from determining the underlying pathogenesis of the patient's cardiomyopathy. In many AF patients, dabigatran or rivaroxaban may both prove to be efficacious in thrombus prevention and treatment. However, some patients may show resistance to NOAC treatment, requiring an alternative therapeutic strategy. While the factors mediating the heterogeneous responsiveness to NOACs are currently unclear, it has important implications for individualized NOAC therapy for thrombus treatment and prevention that warrants further consideration.

## Author contributions

**Conceptualization:** Huan Sun, Qini Zhao, Robert Lakin, Mengjie Yan.

**Data curation:** Xueyan Liu, Ming Yu.

**Funding acquisition:** Yuquan He, Ping Yang.

**Investigation:** Huan Sun, Dongmei Gao, Mengjie Yan.

**Methodology:** Qini Zhao, Yanjing Wang, Dongmei Gao.

**Resources:** Xueyan Liu, Ming Yu, Hongliang Yang, Guangyuan Gao.

**Software:** Qini Zhao, Yanjing Wang, Hongliang Yang, Guangyuan Gao.

**Supervision:** Yuquan He, Ping Yang.

**Validation:** Robert Lakin, Weiwei Chen, Yuquan He.

**Visualization:** Yanjing Wang.

**Writing – original draft:** Huan Sun.

**Writing – review & editing:** Robert Lakin, Guangyuan Gao, Mengjie Yan.
